# Rapamycin and hydroxychloroquine combination alters macrophage polarization and sensitizes glioblastoma to immune checkpoint inhibitors

**DOI:** 10.1007/s11060-019-03360-3

**Published:** 2020-02-04

**Authors:** Sanford P. C. Hsu, Yi-Ching Chen, Hsin-Chien Chiang, Yi-Chun Huang, Cheng-Chung Huang, Hsin-Ell Wang, Yu-Shang Wang, Kwan-Hwa Chi

**Affiliations:** 1 Department of Neurosurgery, Neurological Institute, Taipei Veterans General Hospital, Taipei, Taiwan; 2 School of Medicine, National Yang Ming University, Taipei, Taiwan; 3 JohnPro Biotech Inc., Taipei, Taiwan; 4 Department of Biomedical Imaging and Radiological Sciences, National Yang-Ming University, Taipei, Taiwan; 5 Department of Radiation Therapy and Oncology, Shin Kong Wu Ho-Su Memorial Hospital, #95, Wen-Chang Rd., Shi-Lin, Taipei, 11101 Taiwan (ROC)

**Keywords:** Macrophage polarization, Anti-PD1, Rapamycin, Hydroxychloroquine, Glioblastoma

## Abstract

**Introduction:**

The failure of immune checkpoint inhibitor (ICPi) on glioblastoma (GBM) treatment underscores the need for improving therapeutic strategy. We aimed to change tumor associated macrophage (TAM) from M2 type (anti-inflammatory) to M1 (pro-inflammatory) type to increase the therapeutic response of ICPi. We proposed that combined rapamycin (R) and hydroxychloroquine (Q) preferentially induce M2 cells death, as fatty acid oxidation was their major source of energy.

**Methods:**

Macrophage polarization was characterized on mice and human macrophage cell lines by specific cytokines stimulation with or without RQ treatment under single culture or co-culture with GBM cell lines. Tumor sizes were evaluated on subcutaneous and intracranial GL261 mice models with or without RQ, anti-PD1 mAb treatment. Tumor volumes assessed by MRI scan and proportions of tumor infiltrating immune cells analyzed by flow cytometry were compared.

**Results:**

In vitro RQ treatment decreased the macrophages polarization of M2, increased the phagocytic ability, and increased the lipid droplets accumulation. RQ treatment decreased the expression levels of CD47 and SIRPα on tumor cells and macrophage cells in co-culture experiments. The combination of RQ and anti-PD1 treatment was synergistic in action. Enhanced the intra-tumoral M1/M2 ratio, the CD8/CD4 ratio in the intracranial GL261 tumor model after RQ treatment were evident.

**Conclusion:**

We provide a rationale for manipulating the macrophage phenotype and increased the therapeutic effect of ICPi. To re-educate and re-empower the TAM/microglia opens an interesting avenue for GBM treatment.

**Graphic Abstract:**

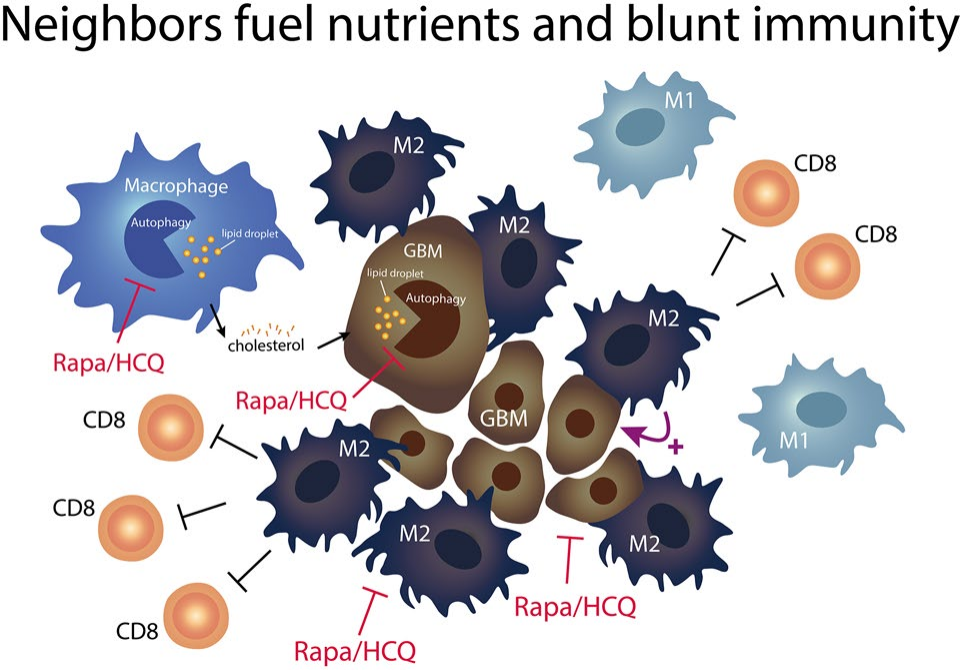

## Introduction

GBM is an aggressive malignancy with high mortality, relatively resist to radiation, chemotherapy and immunotherapy [1–5]. Although the therapeutic effect of ICPi had been tested in various settings, the objective response rate of anti-PD-1 in recurrent GBM patients remained low, and the duration of response was short [6, 7]. To overcome the limitation of ICPi treatment on GBM, it is necessary to change the unfavorable direction inside the tumor microenvironment (TME) that can affect the response. Tumor associated macrophage (TAM) not only inhibit CD8 + T cell immune response against cancer via PD-1/PD-L1 pathway, but also directly inhibit the phagocytes function of macrophages [8]. Glioma associated macrophages (GAM) were GBM specific TAM, which were either transformed from peripheral origin or brain-intrinsic microglia that created a supportive stroma for GBM expansion and invasion [9]. GAM accounted for approximately 30% to 50% of GBM bulk cell populations which may explain the immunosuppressive features of GBM [8, 10].

It has been reported that PD–1 promotes macrophages toward alternative macrophage activation phenotype (M2), and PD-1 expression on macrophage correlates negatively with phagocytic potency [11]. The blockade of PD-1-PD-L1 axis increased macrophage phagocytosis [11]. Reprogramming TAMs by targeting M2 polarization might overcome immune suppression and enhance response rates to anti-PD-1/PD-L1 therapy [12, 13]. We hypothesized that using metabolic modulation strategy to reverse the polarization direction in GAM to trigger phagocytosis might be able to enhance the therapeutic effect of anti-PD-1 [14].

We have reported the combination of autophagy inducer, sirolimus (R) and autophagy inhibitor, hydroxychloroquine (Q) presented synergistic killing effect on GBM cells through the induction of lysosomal membrane permeabilization from cholesterol depletion [15]. The lysosome breaks down macromolecules and is involved in a variety of cellular processes that cholesterol is critical for their stability [16]. GBM cells are characterized by high phagocytosis, lipogenesis, exocytosis activities for nutrients uptake especially cholesterol and other fatty acids from neighborhoods, and high lysosomal demand was necessary for their survival and invasion. Lysosome is also very important on the function of macrophages (microglia) because of their high phagocytic nature [17]. If fatty acid oxidation are their main source of energy supply, then lysosome is on the cross road of autophagy and lipolysis. Therefore, the strategy of using mTOR inhibitor (R) to augment the effect of autophagy inhibition by pushing more autophagy need while blocking the last autophagosome process by Q as a final blow. It will cause energy imbalance and disrupt the formation of M2.

In the present study, we aimed to investigate the ability of combined RQ to enhance the ICPi effect by modulation the component proportion and the function of TAMs. We are the first group reporting RQ may decrease the M2 polarization and increase the ability of macrophage phagocytosis related to down-regulation of CD47-SIRPα axis. Our data provides a rational design of anti-PD-1 and RQ combination therapy for GBM.

## Materials and methods

### Cell culture and macrophage polarization

Macrophage cell lines Raw264.7, J774a.1 and THP-1 were obtained from Bioresource Collection and Research Center (BCRC, Taiwan). GBM cell lines GL261 and GBM8401 were kindly provided by Dr. Hsin-Ell Wang (Department of Biomedical Imaging and Radiological Sciences, National Yang-Ming University, Taiwan). All mediums and supplements were purchased from ThermoFisher Scientific (MA, USA). Macrophage polarization protocol was as follows: Human THP-1 monocytes were differentiated into macrophage by incubation in the presence of 100 ng/mL of PMA (phorbol 12-myristate 13-acetate) (Sigma-Aldrich, MO, USA) for 16 h. Cells became adherent and defined as M0. These M0 cells were then polarized into M1 macrophages by incubation with M1-inducer, LPS (50 ng/mL) and IFN-γ (50 ng/mL), for 48 h; polarized into M2 macrophages by incubation with M2-inducer, IL-4 (50 ng/mL) and IL-13 (50 ng/mL), for 48 h. The mouse macrophage cell lines Raw264.7 and J774a.1 were polarized into M1 by incubation with M1-inducer, IFN-γ (100 ng/mL), for 48 h; polarized into M2 by incubation with M2-inducer, IL-4 (50 ng/mL) for 48 h. All inducers were purchased from PeproTech (NJ. USA). The polarized macrophages were stained with antibodies against CD80 (PerCP/cy5.5-conjugated, 305231), CD163 (APC-conjugate, 333610); tumor infiltrating lymphocyte was stain with antibodies against CD86 (Alexa Fluor 647-conjugated, 105020), CD206 (PE-conjugate, 141706), CD4 (PE-conjugate, 116006), CD8 (PE-conjugate, 100708), F4/80 (FITC-conjugate, 123108), and CD45 (PerCP/cy5.5-conjugated, 103132) for 30 min at 4 °C, and washed twice by FACS buffer (1% FBS in PBS) and analyzed by flow cytometry. Cells were treated with R (3 μM) and Q (9 μM) for 48 h. All antibodies were purchased from BioLegend (CA, USA).

### Western blot analysis

For protein analysis, blots were developed using ECL chemiluminescent detection system (GE Life Science, Buckinghamshire, UK). p-STAT1 (#7649), p-STAT6 (#56554), Arginase 1 (#93668), and β-tubulin (#2128) were purchased from Cell Signaling (MA, USA). CD47 (GTX53912), SIRPα (GTX112645) were purchased from GeneTex (CA, USA). iNOS (ab3523) was purchased from Abcam (Cambridge, UK),

### Quantitative real-time PCR

Total RNA was extracted using RNeasy Mini Kit (Qiagen, Hilden, Germany) according manufacturer’s protocol. After reverse transcription using cDNA reverse transcriptase (ThermoFisher, MA. USA) and Oligo(dT) primer, quantitative real-time PCR (qRT-PCR) was performed using SYBR Green PCR Mix and IQ5 detection system (Bio-Rad, CA, USA). mRNA expression was normalized to β-actin, data are presented as relative quantification of M0 control based on the calculation of 2^−∆∆Ct^.

### Lipid staining and bio-function analysis of macrophage

After polarization, Raw264.7 cells were incubated with 2 μg/ml BODIPY 493/503 (Molecular Probes, OR, USA) for 15 min at 37 °C. Cells were washed by FASE buffer and analyzed by flow cytometry. Polarized Raw264.7 cells were divided into three groups: control group in 37 °C, FITC-dextran in 4 °C group, and FITC-dextran in 37 °C group, with each containing 2 ~ 3 × 10^5^ cell in 200 μL medium. Cells were added to 2 μL FITC-dextran and incubated in either 4 °C or 37 °C for 20 min, and then chilled on ice immediately. Fluorescence intensity was analyzed by flow cytometry.

### Mouse GL261 glioma xenograft model

Female C57BL/6 J mouse (20-22 g, 6-8 weeks) purchased from the BioLASCO Taiwan Co., Ltd. All procedures were performed according to the guidelines approved by the Animal Care and Use Committee of the National Yang-Ming University. Mouse GL261 glioma cells (4 × 10^6^) in 0.1 ml of phosphate-buffered saline (PBS) were injected subcutaneously into the right flank of the C57BL/6 mice. When tumor growth reached 200 mm^3^, mice were randomly distributed into four groups (n = 6 per group): Control group, RQ group (rapamycin, hydroxychloroquine, Sigma), Anti PD-1 mAB group (anti PD-1 monoclonal antibody) treatment group (RMP1-14, BioXCell), and Combined group (RQ plus anti PD-1 mAb). The dose of anti PD-1 mAb was 200 µg/mice, intraperitoneal injection (i.p.), every 2 days for a total of three times. The dose of rapamycin was 5 mg/kg and chloroquine was 50 mg/kg, i.p., for 14 consecutive days. In orthotropic GL261 model, female 6 to 8 week-old C57BL/6 J mice were anesthetized via i.p. administration of pentobarbital at 40 mg/kg body weight. Their heads were shaved above the nape of the neck, scrubbed with Betadine/alcohol, and immobilized in a Cunningham Mouse/Neonatal Rat Adaptor stereotactic apparatus (Stoelting, Wood Dale, IL, USA). A 5 mm skin incision was made at the sagittal suture, then a burr hole was created, and 2 × 10^5^ GL261 cells in 2 μl of culture medium was injected stereotactically into a single defined left hemisphere location (0.14 mm anterior and 2.0 mm lateral to the bregma) of each mouse brain at 3.5 mm depth. Three days following the tumor implantation, the mice were randomly distributed into four groups (n = 6 per group) and given same treatment protocol and dose as subcutaneous GL261 model (Fig. 5a).

### Magnetic resonance imaging (MRI) and tumor measurement

MRI of anesthetized tumor-bearing animals was performed with a Bruker PET/MR 7T system (Bruker, Germany). All images were obtained as follows: The T2-weighted fast spin echo (FSE) sequence images were acquired with a 256 × 256 × 25 matrix (X, Y, and A), Pixel size was 0.0781 × 0.0781 mm, Thickness was 0.5 mm, FOV = 20 mm, Echo: 33.0 ms, repetition: 2654.7381. The DICOM images were collected, and the tumor boundary visualized in each slice was contoured and the tumor volume was calculated by treatment planning system Pinnacle3 9.8 (Philips Radiation Oncology System, Fitchburg, WI).

### Statistical analysis

All statistical analysis was performed using Prism 5 (GraphPad, La Jolla USA). The experimental and control groups were compared using an unpaired, two-tailed, Student’s *t* test. Statistical analysis was performed at the P < 0.05 and P < 0.01 (denoted as * and **).

## Results

### Macrophage polarization altered towards M1-like by RQ treatment

**Fig. 1:**
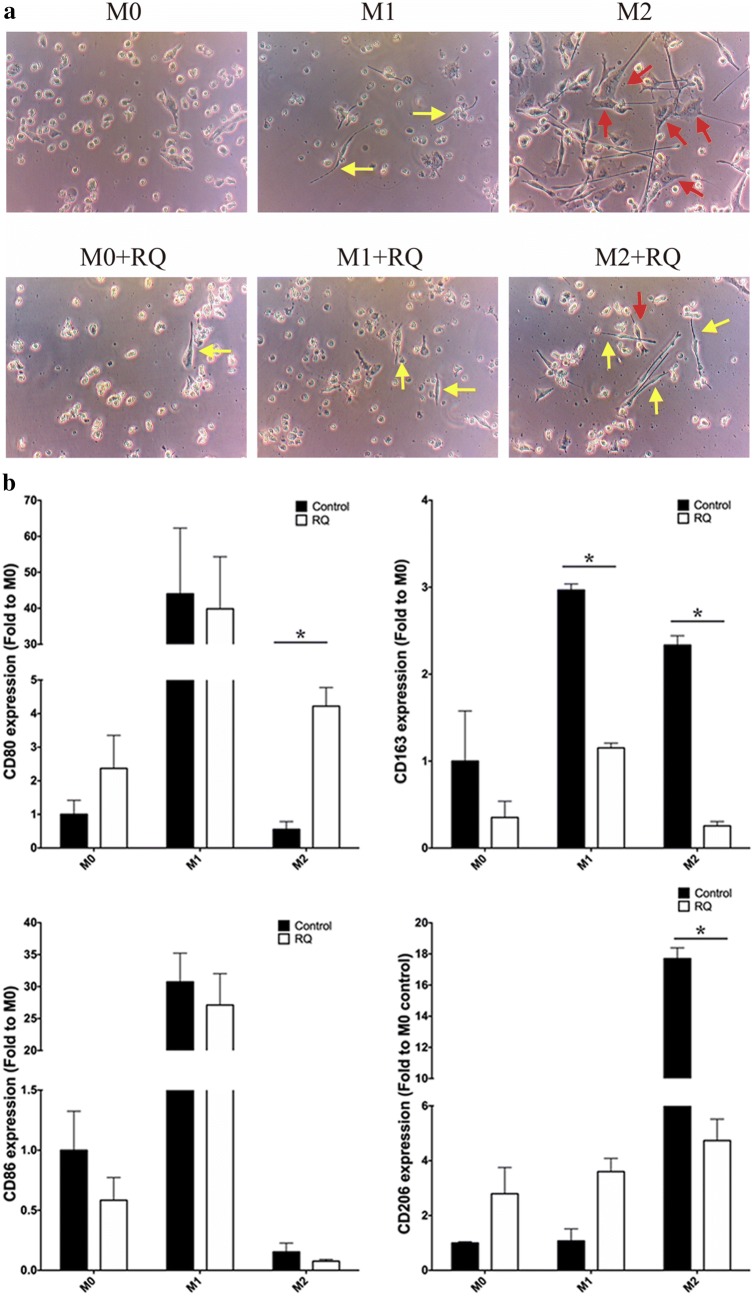
Macrophage polarization altered towards to M1-like by RQ treatment. **a** After PMA treatment for 16 h, THP-1-derived macrophage was polarized with M1-inducer (LPS, IFN-γ) or M2-inducer (IL-4, IL-13) with or without RQ for 6 days. The M0 cells exhibit as the round shape, M1 cells as the spindle-shaped (yellow arrow), and M2 cells with spread-filopodia shape (red arrow). All three types of macrophages showed M1-like morphology after RQ treatment. **b** Flow cytometry analysis of M1 surface markers CD80, CD86 and M2 markers CD163, CD206 on THP-1-derived and J774a.1macropaghe, respectively. Both cell lines showed significantly decreased expression of M2-related markers in M2 + RQ group versus the M2 group (P < 0.05). (Upper panel: THP-1-derived macrophage. Lower panel: J774a.1 macrophage)

Figure 1a shows the morphology after 6 days of incubation. M1 has spindle-shaped morphology (yellow arrow), M2 exhibited a more spread filopodia shape (red arrow), and M0 as round-shaped. With RQ treatment during polarization, all three types of macrophages (M0, M1, and M2) showed increased numbers of M1-like morphology (spindle shaped). Flow cytometry analyzed the M1-surface marker, CD80 and CD 86 and the M2-surface markers, CD163 and CD206, on THP-1 and J774a.1cells, respectively. Both cell lines showed significantly decreased expression in the M2 + RQ group versus the M2 group (P < 0.05) (Fig. 1b). These results indicate macrophage polarization can be altered by RQ treatment, resulting in M1-like morphology.

### RQ treatment decreased M2-related phenotypes

**Fig. 2:**
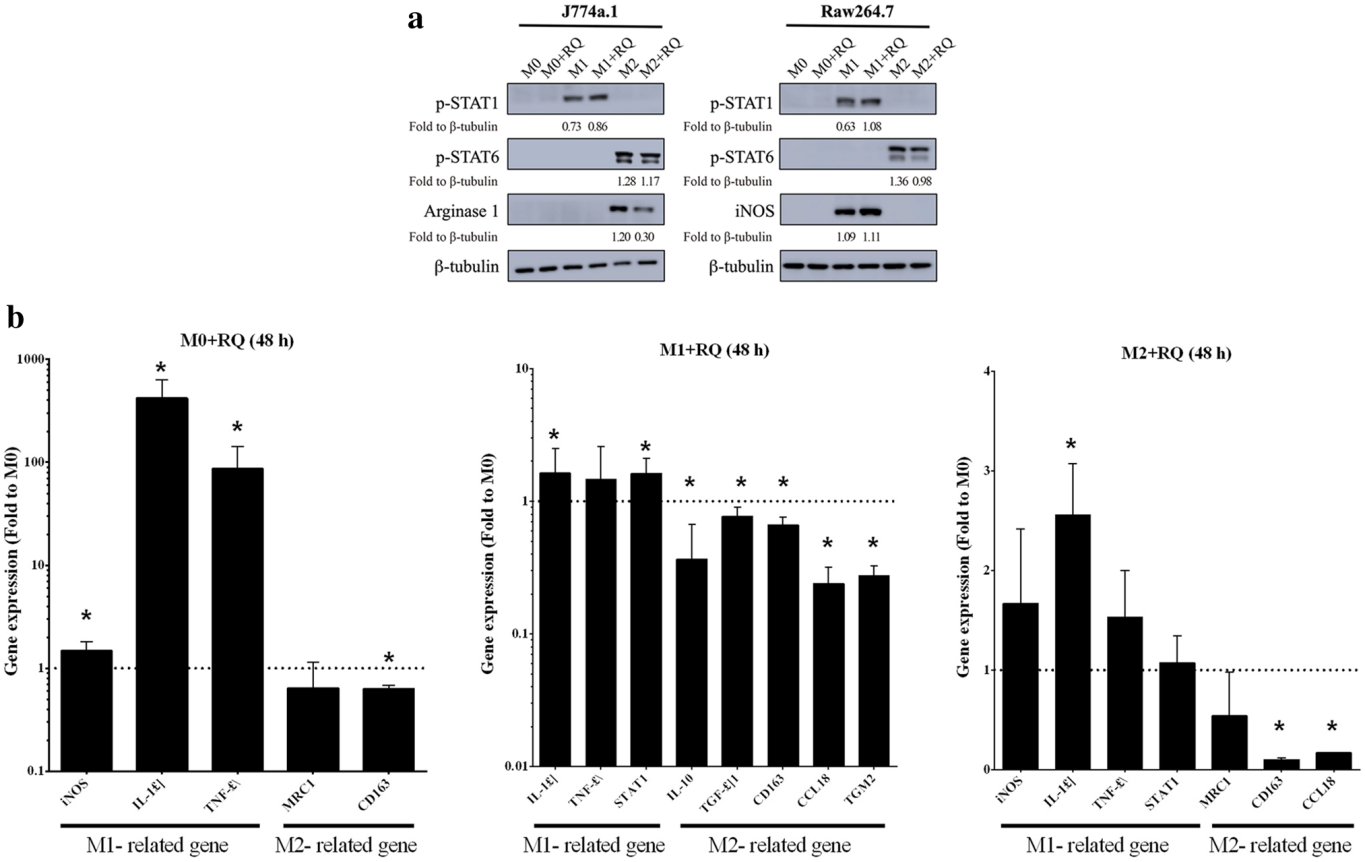
RQ treatment decreased M2-like phenotypes. **a** J774a.1 and Raw264.7 cells were each divided into six groups, M0, M1, M2, M0 + RQ, M1 + RQ, and M2 + RQ. p-STAT1 and iNOS was found to be upregulated in M1 and M1 + RQ groups. p-STAT6 and arginase-1 was downregulated in M2 and M2 + RQ groups. **b** Real-time PCR showed increased expression of M1-related genes in M0 cell and decreased expression of M2-related genes in M2 cells after RQ treatment compared with M0 baseline control

Western blot was used to detect protein expressions related to macrophage polarization. Previous studies [18] have shown IFN-γ to activate STAT1 and induce expression of M1-associated genes, such as iNOS; IL-4 and IL-13 has been shown to activate STAT6 and induce expression of M2-associated genes. We cultured J774a.1 and Raw264.7 with M1-inducer, and found phospho-STAT1 to be upregulated, which was further increased when RQ was present (P < 0.05). In J774a.1 and Raw264.7 cultures, phospho-STAT6 was found to be increased in the M2-inducer group, and downregulated in the M2 + RQ group (P < 0.05), also noted with arginase-1 in J774a.1 cell (P < 0.05) (Fig. 2a). Real-time PCR was used to analyze M1 and M2-related gene expression profile in M0 + RQ, M1 + RQ, and M2 + RQ, using M0 as baseline control. In the M0 + RQ group, M1-related genes, IL-1β and TNF-α were upregulated, while the M2-related genes MRC1 and CD163 were downregulated. In the M1 + RQ group, M1-related genes IL-1β, TNF-α, and STAT1 were upregulated, while M2-related genes IL-10, TGF-β1, CD163, CCL18, and TGM2 were downregulated. In the M2 + RQ group, M1-related genes iNOS, IL-1β, TNF-α, and STAT1 were upregulated, while M2-related genes MRC1, CD163, and CCL18 were downregulated (Fig. 2b). These data indicate RQ increased M1-related gene expression and decreased M2-related gene expression.

### RQ treatment increased phagocytosis ability of M0 and M2 Macrophages

**Fig. 3:**
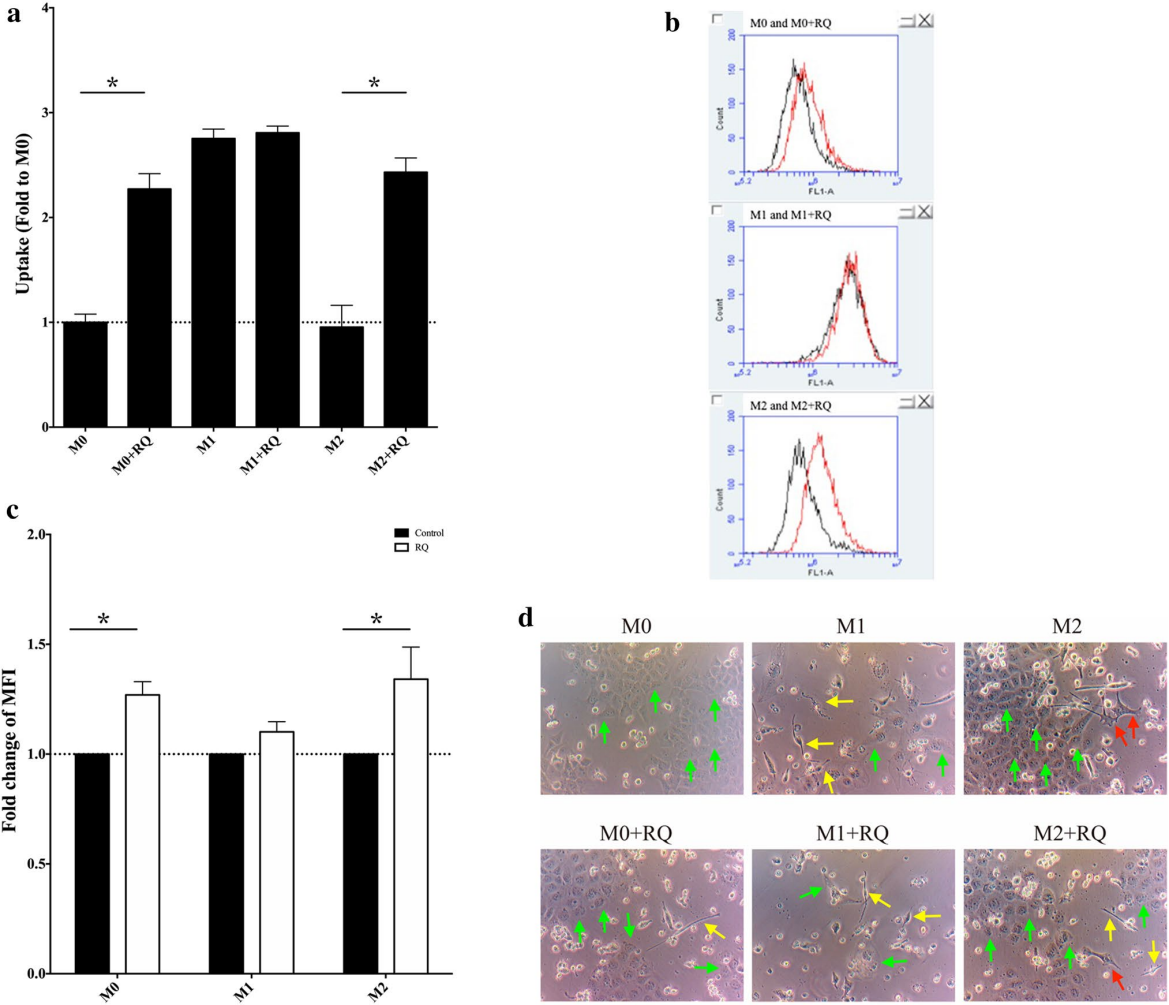
RQ treatment increased phagocytosis ability of M0 and M2 macrophages. **a** Flow cytometry of Dextran-FITC showed RQ significantly increased phagocytosis uptake in M0 + RQ and M2 + RQ groups versus non-RQ-treated groups. Both M1 and M1 + RQ groups showed high uptake rate without difference. **b** and **c** BODIPY staining of Raw264.7 showed an increased lipid droplet accumulation in M0 + RQ and M2 + RQ groups versus non-RQ-treated groups. *MFI* Mean fluorescence intensity. **d** CFSE-positive GBM8401 cells were co-cultured for 6 days with THP-1 macrophage under different activation conditions. GBM cells were decreased under M1 culture condition and significantly decreased in M0 and M2 cells with or without RQ treatment (P < 0.05). Green arrow indicates GBM8401 cancer cells. Yellow arrow indicates M1-like cells (spindle-shaped). Red arrow indicates M2 cells (spread filopodia)

Cultured Dextran-FITC with Raw264.7 cells showed the M0 and M2 groups had inherently low uptake ability, when treated with RQ, the M0 + RQ and M2 + RQ groups showed significant increase in phagocytosis uptake (P < 0.05) (Fig. 3a). It has been reported the macrophage phagocytosis was correlated with the accumulation of lipid droplet [19]. BODIPY-staining was used to analyze lipid accumulation by flow cytometry in six experiment groups. M0 + RQ and M2 + RQ showed increased lipid droplet accumulation versus non-RQ treatment groups (Fig. 3b, c). CFSE-positive GBM8401 cells were co-cultured for 6 days with THP-1 macrophage. Both M1 and M1 + RQ groups showed strong ability to kill tumor cells, while M1-like macrophages (yellow arrow) were increased in all of the RQ-present groups (Fig. 3d).The increased phagocytosis ability of M0 and M2 macrophages indicate RQ may have the ability to alter M0 and M2 to M1-like.

### RQ treatment decreased CD47 and SIRPα expression

**Fig. 4:**
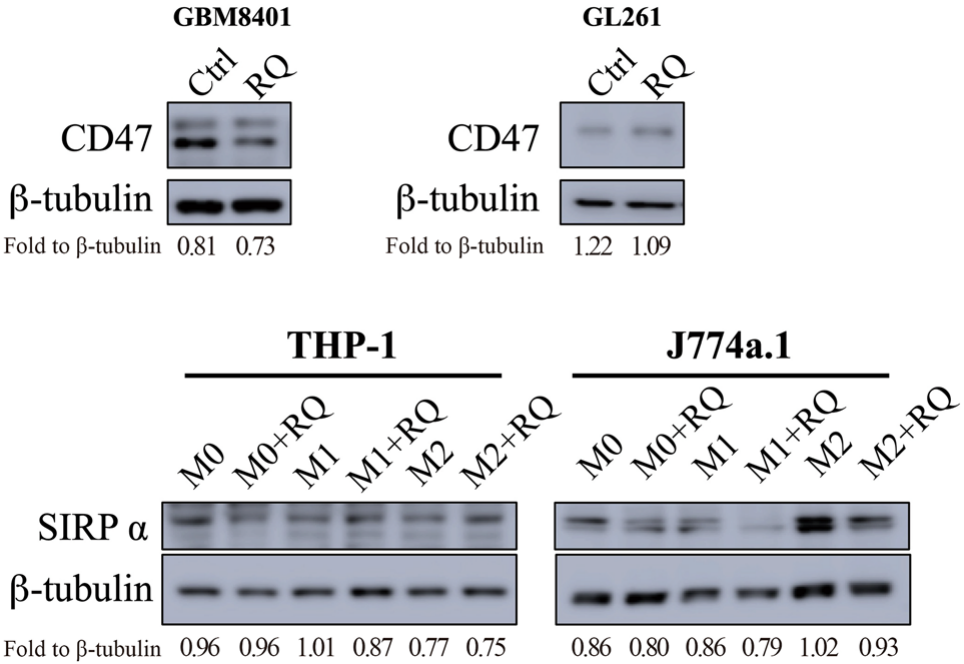
RQ treatment decreased CD47 and SIRPα expression. Western blotting of GBM8401 and GL261 cancer cells co-cultured with THP-1 and J774a.1 with or without RQ treatment. It showed that CD47 to be downregulated in GBM8401 cells, while SIRPα expression was downregulated in J774a.1 cells after RQ treatment

Over the course of glioma-macrophage co-culture experiments, we found interesting results with CD47 and SIRPα expression. When GBM8401 and GL261 cancer cells were cultured with RQ for 48 h, CD47 was downregulated. Meanwhile, SIRPα expression was also downregulated in THP-1-derived and J774a.1 macrophages (p value not reached) (Fig. 4). Our datasuggest that the tumor killing ability of RQ-present co-culture may be linked to the prevention of phagocytosis-inhibition resulting from CD47-SIRPα interaction.

### In vivo efficacy evaluation of combined RQ and anti-PD-1 treatment in GL261 xenograft models

**Fig. 5:**
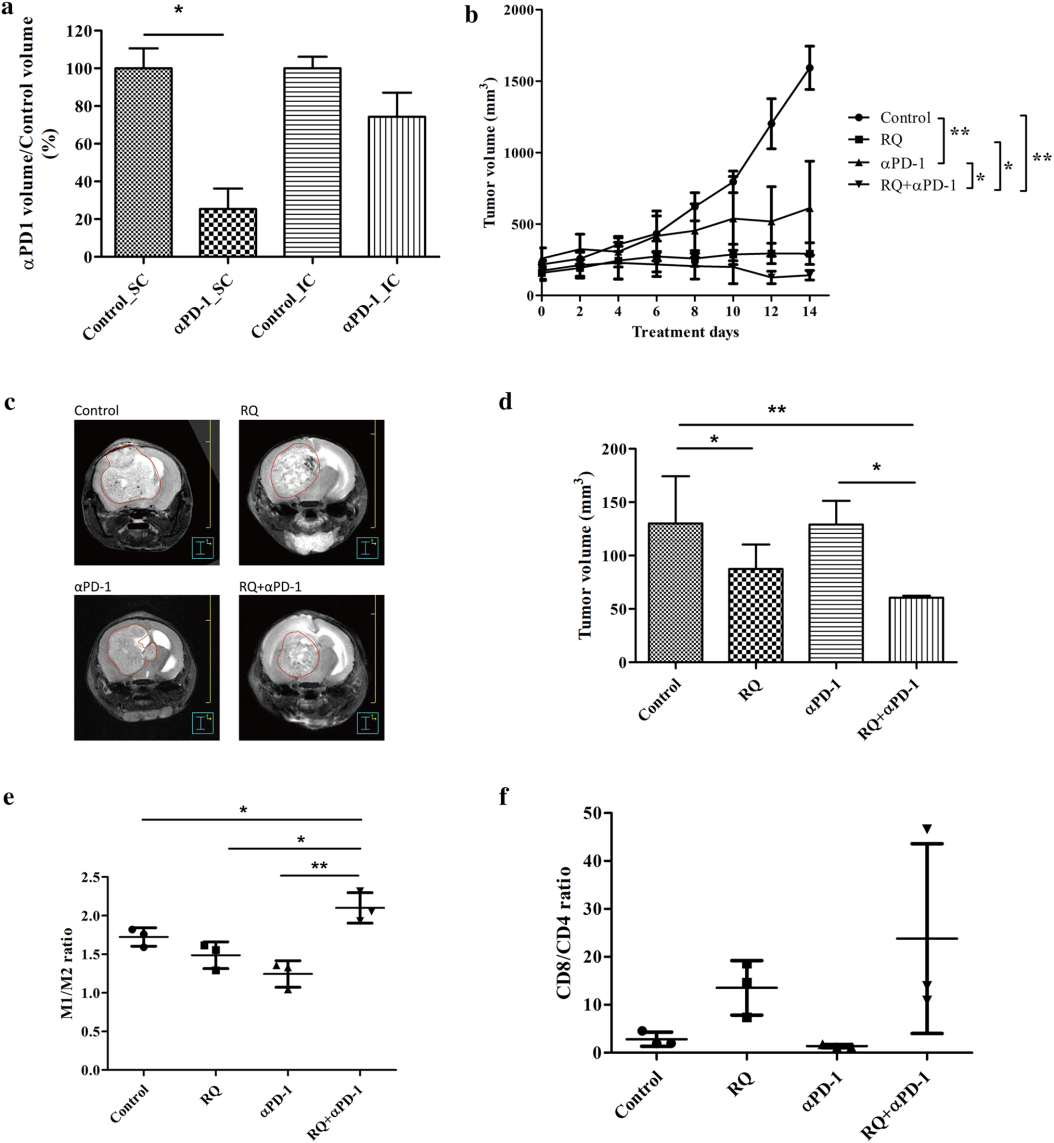
In vivo efficacy evaluation of combined RQ and anti-PD1 treatment in GL261 xenograft models. **a** Comparison of anti-PD-1 inhibition of tumor growth in subcutaneous (SC) and intra-cranial (IC) GL261 mice models. IC tumor was not as effective as SC tumor to anti-PD-1 treatment. **b** Growth inhibition curve of SC GL261 tumor model. Each data point represents the average tumor burden of 6 mice. **c** The representative photograph of T2-weighted FSE MRI scans of orthotropic GL261 intra-cranial tumor model. MRI images of GL261 were acquired 16 days following IC implantation of 2 × 10^5^ GL261 cells. The tumor size of each group of tumor-bearing mice was assessed by 7T micro PET/MRI T2-weighted FSE imaging (n = 4 mice/group). **d** Volumetric assessment of the glioma in different treatment groups (n = 4). (Error bars, mean ± SEM; *P < 0.05, **P < 0.01). GL261 orthotropic mice were sacrificed on 14th day for all treatment groups and flow cytometry was used to analyze **e** M1 to M2 ratio, **f** ratio of CD8 to CD4 T cells (n = 4). (Error bars, mean ± SEM; *P < 0.05, **P < 0.01)

The efficacy of anti-PD-1 treatment on subcutaneous (SC) or intracranial (IC) tumor models are summarized in Fig. 5a. IC tumors are less responsive to ICPi treatment, which might suggest more immunosuppressive in the brain TME. Although RQ combined with anti-PD-1 treatment did significantly delay the SC tumor growth than anti-PD-1 alone (Fig. 5b), but not as obvious as that in IC model (129.10 ± 38.25 mm^3^ versus 60.63 ± 2.97 mm^3^, P < 0.05, n = 4 mice/group) (Fig. 5c, d). Flow cytometry analysis of tumor infiltrating immune cells was then performed. The results showed that the ratio of M1 and M2 in the combined treatment group (2.10 ± 0.20) was significantly higher than control group (1.79 ± 0.18, P < 0.05), RQ group (1.4 ± 0.22, P < 0.05), and anti-PD-1 group (1.51 ± 0.56, P < 0.01) (Fig. 5e). We found a trend of increased CD8/CD4 in RQ combined with anti-PD-1treatment (Fig. 5f).

## Discussion

We demonstrated that anti-PD-1 treatment resulted in significant growth delay in SC GL261 mouse model, while only modest effectiveness in IC model. It suggested that brain-resident microglia might play a specific immunosuppressive role in the response to anti-PD1 treatment in GBM. RQ treatment impairs the polarization of M2 macrophage through jeopardizing lipid utilization. RQ increased the phagocytic ability of macrophages and decreased the in vitro expression levels of CD47 and SIRPα on tumor cells and macrophages, respectively. This may unleash the phagocytosis inhibitory function through CD47/SIRPα interaction. The combination of RQ and anti-PD-1 treatment enhanced the intra-tumoral M1/M2 ratio, the CD8/CD4 ratio and the phagocytic ability. Hence, to re-educate and re-empower the TAM/microglia opens an interesting avenue for GBM treatment.

Characterized phenotypes (morphologic, genotypic) were seen in different polarized macrophages, which contributed to their activation state. We showed in Figs. 1 and 2 that M1 polarized macrophage cells looked smaller, minimally branched compared with M2 polarized cells, which were larger, highly branched cells. RQ treatment turned the morphology of highly branched M2 cells into more small rounded M1 cells, and significantly down-regulated the expression of M2-like macrophage markers (CD163, CD206, Arginase 1, pSTAT6). M1-like molecules (CD80, CD86, pSTAT1, iNOS) were not changed by RQ treatment. Although the classical assumption model suggested a rigid dichotomy between iNOS-positive macrophage (M1) and Arginase 1-positive macrophage (M2) were not unequivocally distinct between different cell lines under same cytokines stimulation, our in vitro result did demonstrate that RQ reduced the polarization of M2-like macrophages. Under macrophage-glioma co-culture system as shown in Fig. 3d, RQ treatment significantly reduced the GBM cells and the proportion and number of M2 type cells. As shown in Fig. 5c, d, RQ plus anti-PD-1 treatment significantly reduced the IC tumor volume. The volume assessed by MRI scan was not only quantitative, but also characteristic that multiple small high signal intensity necrotic changes from T2-weighted FSE sequence images after RQ treatment. The combined treatment significantly increased the intra-tumoral M1/M2 ratio and especially the CD8/CD4 ratio (Fig. 5e, f). Conceivably, with immune checkpoint inhibition combined with RQ treatment might lead to a synergistic response benefit in mice with established brain tumors.

The widely accepted anti-tumor mechanism of PD-1/PD-L1 blockade is rejuvenating T cells [20]. Macrophages possess intrinsic tumoricidal activity, thus the PD-1 blockade would induce both M1 macrophage polarization and increases macrophage phagocytosis [11, 21]. Microglia have strong phagocytic capacity, which can be either neuroprotective or neurotoxic by different microenvironment stimuli [22]. The microglia was polarized into tumor supportive and immune suppressive phenotype in the milieu of GBM [23]. TAMs within the glioma tended to be pro-tumorigenic and associated with tumor grade had long been reported [23, 24]. TME in brain may limit the reaching of T cells into tumor and reduced the viability of tumor infiltrating lymphocytes [25]. Recently, Hutter et al. had again reported that microglia distinctly showed lack of inflammatory response [8].

The foamy appearance of PD-1 + TAMs showed accumulation of un-cleared phagocytic materials, which suggested the vulnerability of lysosomes in this type of cells [11]. Increased aerobic glycolysis was observed in M1 macrophages, whereas fatty acid oxidation was observed in M2 macrophages [26]. Inhibition of fatty acid oxidation blocked M2 activation [27]. It would be interesting to note from Fig. 3b, c that lipid droplets were accumulated after RQ treatment. In addition, a recent study identified cell-intrinsic lysosome lipolysis as a critical source of fatty acids that fuel the M2-like response process [28]. Based on our previous observation that GBM cells can only survive from high lipolysis under stress. They could not uptake cholesterol from serum because of blood brain barrier. The cholesterol and fatty acids need coming from lipolysis or phagocytosis from neighborhoods [15]. Goossens et al. had recently reported that ovarian cancer cells promote membrane cholesterol efflux and depletion of lipid rafts from neighbor macrophages and therefore drives the TAM reprogramming [29]. We hereby showed the lipid metabolism in TAM might be changed with lipid droplets accumulation after RQ treatment. We found RQ may decrease the expression levels of CD47 and SIRPα on tumor cells and macrophages, respectively. Our data echoes the observation from Zhang et al. that combined agents disrupting CD47-SIRPα axis and autophagy inhibition elicit stronger anti-tumor effect on GBM cells [30]. One limitation of present study is that we have not examined the individual role of microglia and monocyte derived macrophages by genetically color-coded experiments. Further deciphering mechanisms on cholesterol and other fatty acid metabolism are needed.

## Conclusions

The experimental results presented here clearly show that RQ promotes TAM polarization toward a more pro-inflammatory M1-like phenotype, down-regulate tumor CD47-SIRPα axis and improve the ICPi effect through macrophage. Co-targeting tumor and macrophages with RQ through lipid homeostasis reveals a promising therapeutic strategy in GBM.
